# A Model of Repetitive-DNA-Organized Chromatin Network of Interphase Chromosomes ^†^

**DOI:** 10.3390/genes3010167

**Published:** 2012-03-07

**Authors:** Shao-Jun Tang

**Affiliations:** Department of Neuroscience and Cell Biology, University of Texas Medical Branch, Galveston, TX 77555, USA; E-Mail: shtang@utmb.edu; Tel.: +1-409-772-1190

**Keywords:** repetitive DNA, repetitive element, repetitive sequence, DNA repeat, junk DNA, transposons, chromosome, chromatin

## Abstract

During interphase, chromosomes are relatively de-condensed in the nuclear space. Interphase chromosomes are known to occupy nuclear space in a non-random manner (chromosome territory); however, their internal structures are poorly defined. In particular, little is understood about the molecular mechanisms that govern the internal organization of interphase chromosomes. The author recently proposed that pairing (or interaction) of repetitive DNA-containing chromatin regions is a critical driving force that specifies the higher-order organization of eukaryotic chromosomes. Guided by this theoretical framework and published experimental data on the structure of interphase chromosomes and the spatial distribution of repetitive DNA in interphase nuclei, I postulate here a molecular structure of chromatin organization in interphase chromosomes. According to this model, an interphase chromosome is a chromatin mesh (or lattice) that is formed by repeat pairing (RP). The mesh consists of two types of structural components: chromosome nodes and loose chromatin fibers. Chromosome nodes are DNA repeat assemblies (RAs) that are formed via RP, while loose fibers include chromatin loops that radiate from the nodes. Different loops crosslink by RPs and form a large integrated chromatin network. I suggest that the organization of the chromatin network of a given interphase chromosome is intrinsically specified by the distribution of repetitive DNA elements on the linear chromatin. The stability of the organization is governed by the collection of RA-formed nodes, and the dynamics of the organization is driven by the assembling and disassembling of the nodes.

## 1. Introduction

The importance of chromatin organization in controlling genome activity is undisputed [1]. It is an emerging consensus that, during interphase, individual chromosomes occupy non-random territories in the nucleus [2]. Many studies have been performed to characterize the ultrastructure [1,3,4,5,6] and chromatin interactions [7,8] of interphase chromosomes, but the molecular organization of chromatin during interphase is largely unknown.

I have recently proposed a DNA-repeat-based principle for chromatin organization, and named it CORE (chromatin organization by repetitive elements) [9]. According to this hypothesis, chromatin regions with repetitive DNA in the same family tend to associate with one another and form repeat assemblies (RAs). This homologous repetitive-DNA-directed clustering of chromatins was termed repeat pairing (RP). As a result of RP, chromatin regions that are separated in linear genomes can be spatially associated. Thus, the formation of RAs generates a molecular driving force to fold and organize chromatins within chromosomes. In this way, repeats function as chromatin organizer modules. Based on this principle and the spatial organization of DNA repeats, I have postulated a RA-formed skeleton of mitotic chromosomes [10]. It is important to note that the principle of repeat-directed chromatin organization that I have put forward is based on only one assumption, which is RP. Details of RP remain to be worked out, and divergent mechanisms mediated by distinct macro-molecules such as proteins and RNA (e.g., siRNA and RNAi machinery [11]) may be involved. Given the extensive experimental data that appear to support this assumption [9], I feel sufficient confidence in the general correctness of RP. Based on the idea of RP and the supporting experimental evidence, I will discuss here some of the conclusions that can be made about the internal structure of chromatin organization in interphase chromosomes. This structure, if correct, reveals a molecular organization of the chromatin in the seemingly amorphous interphase chromosomes. This chromatin organization may contain considerable information about the functional regulation of interphase chromosomes.

## 2. A Model of Repetitive-DNA-Organized Chromatin Mesh in Interphase Chromosomes

**Figure 1:**
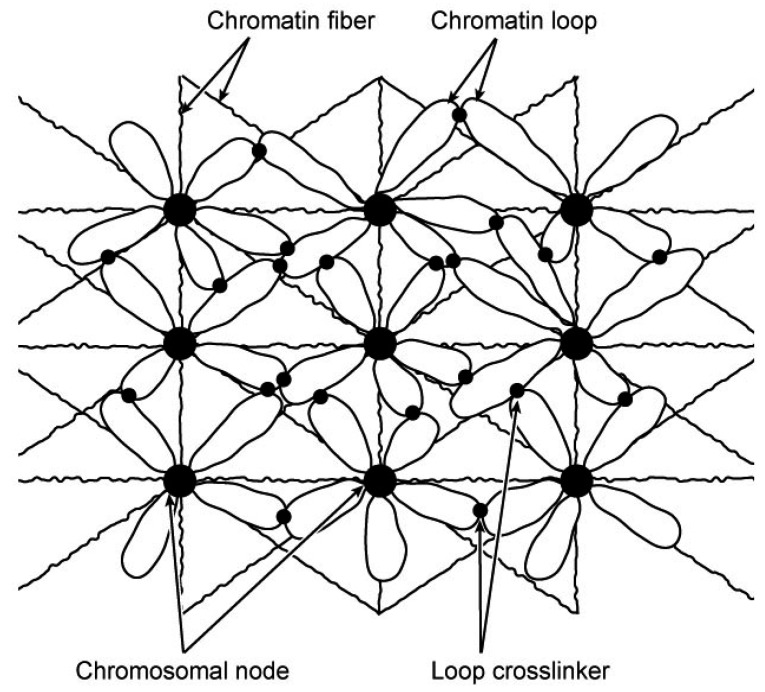
A model of chromatin mesh in interphase chromosomes. According to this model, an interphase chromosome is an integrated chromatin mesh. The mesh is organized by repeat assemblies (RA)-formed chromosomal nodes (bigger dots), from which chromatin loops and fibers emanate. Adjacent chromatin loops may be cross-linked by repeat pairings (RPs) (smaller dots).

**Figure 2:**
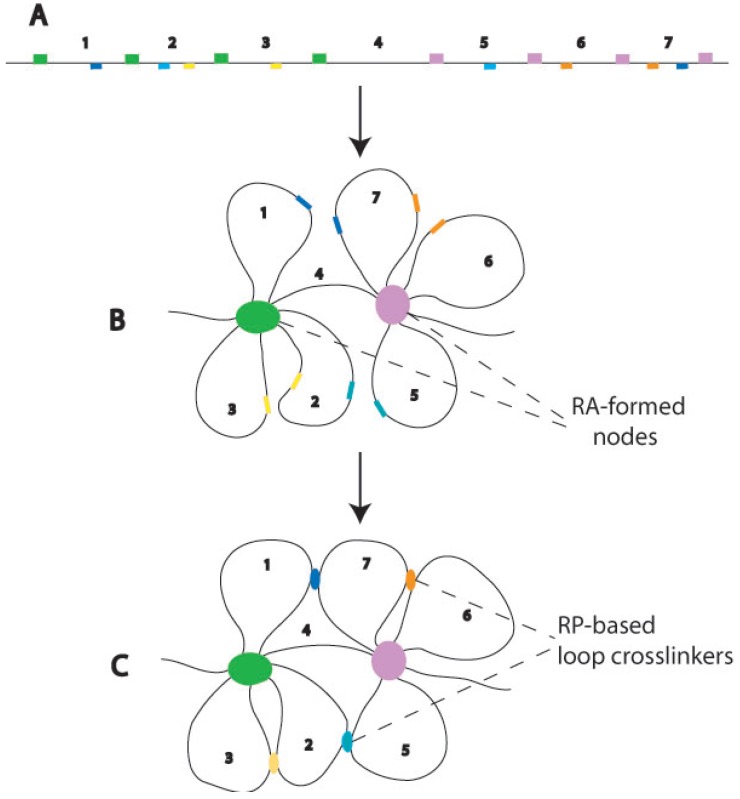
A potential mechanism of RP-directed chromatin mesh formation. (**A**) A hypothetical piece of linear chromatin. Repetitive DNA elements in the same family are indicated by blocks in the same color. Node-forming repeats are depicted above the line while repeats contributing to loop crosslinking below the line. Numbers are used to indicate the intervals between node-forming repeats; (**B**) The formation of chromosomal nodes and chromatin loops, caused by RPs among node-forming repeats (green and purple). As a result, some of the homologous repeats that are far apart in the linear chromatin are brought to the spatial vicinity of one another on different loops (e.g., light and dark blue repeats), and thus their RPs are facilitated; (**C**) RPs among repeats on different loops crosslink the loops into an integrated mesh. The diagrams have been prepared for conceptual illustration; no attempt has been made to accurately depict the details of the structure. Although only intrachromosomal RPs are depicted in this diagram, interchromosomal RPs are also possible to occur in the intermingling regions of different chromosome territories of the ICN model [12].

The basic assumption used to construct this model was that RP-based RA formation drives chromatin organization in the interphase nucleus. The proposed interphase chromosome structure is a chromatin lattice interlocked by RA nodes (Figure 1). Two basic structural elements are in the lattice. One is the RA-formed chromosome nodes; the other is the chromatin fibers that emanate from and interconnect the nodes. RA nodes and loose chromatins around the nodes are the basic structural elements in the lattice. The mesh formation of interphase chromosomes likely involves hierarchical organizational events mediated by RPs (Figure 2). First, RPs among dispersed homologous repeats lead to the formation of RAs and folding of chromatins into loops around RAs. Then, RPs among homologous repeats on different loops (either on the same or different RA nodes) lead the formation of integrated chromatin mesh. The chromatin organization proposed here is consistent with the structural descriptions of interphase chromosomes that have been summarized in the lattice model [4] and the interchromatin network (ICN) model [12]. Importantly, the model described here illustrates a specific molecular mechanism (RP) for the formation of such a chromatin mesh or network organization.

In this model, chromosome nodes are compact chromatin structures of RA complexes that contain DNA repeats and organizational proteins such as “scaffold” proteins (e.g., condensins, TOPO II). The RA nodes function as chromatin crosslinking points for interacting chromatin fibers (Figure 1). Chromatin-chromatin interactions in RA nodes are mediated by RPs rather than knotting. As described before [9], there are three possible types of nodes. One is formed by dispersed repeat assemblies (DRAs), another by tandem repeat assemblies (TRAs), and the third by the association of TRAs and chromatins that have sequences homologous to the TRA-forming tandem repeats (e.g., SARs/MARs and satellite DNA, respectively). Evidence for RPs and RAs have been summarized previously [9,10]. Previous studies observed dense chromatin domains (CDs) in the interphase nucleus [5,13]. CDs were considered as the potential basic structural units of the higher order chromatin organization, according to the chromosome territory-interchromatin compartment (CT-IC) model [1,2]. Although direct evidence is to be documented, it is tempting to conceive a structural coincidence between the observed CDs and the RA nodes proposed here. RA nodes are a unique feature of the model. Because of the compact nature of the proposed nodes, it might be possible to separate them from the bulk chromatins for molecular characterization. The existence of RA nodes formed by specific DNA repeats may be tested by the recently developed technology of chromosome conformation captures [14]. To use the conformation capture approach for this purpose, one major issue needs to be addressed. Because identified sequences need to be mapped back to the genome, it is a prerequisite to know the genomic position of repetitive sequences in order to determine their “associating” partners. Unfortunately, many repetitive and nonprotein-coding sequences are not annotated in the sequenced genomes and thus have been excluded from the current conformation capture analysis.

The structure and function of chromosome nodes suggested here resemble the nodes in the structure of mitotic chromosomes that I proposed [10], but the spatial distribution of nodes differs in the two forms of chromosomes.

I envision that DRA nodes are mainly positioned in the nucleoplasm. They are the major organizers of the chromatin mesh. Relatively loose chromatin fibers surround the RA nodes. Some fibers emanate from the nodes to form loops, while others connect different nodes to form the lattice (Figure 1). On the other hand, TRAs are mainly tethered to the inner peripheral areas of the nucleus. In support of this suggestion, DNA isolated from the nuclear envelope is enriched with highly repetitive sequences [15]. Previous studies have also suggested that heterochromatins, which mainly contain tandem DNA repeats, are associated with the envelope [16,17,18]. TRAs are connected with the chromatin mesh by the formation of tandem-dispersed repeat assemblies (T-DRAs) via RPs [9]. The spatial organization of RAs in the nucleus determines the architecture and the morphology of an interphase chromosome. In other words, similar to what has been suggested for mitotic chromosomes [10], RAs function as the skeleton of interphase chromosomes. An RA-organized interphase chromosome is an integrated chromatin network in the chromosomal territories. RP-based interactions of chromatins emanating from different nodes create a tension that maintains the morphology of interphase chromosomes. RA-based nodes are probably the structural points through which interphase chromosomes interact with nuclear structures such as the matrix. Indeed, previous studies have revealed that the nuclear matrix is associated with repeated DNA [19,20].

The novel feature of the model proposed here is that it identifies RA formation as a key molecular mechanism for establishing the highly cross-linked chromatin lattice of interphase chromosomes. This mechanism, if correct, indicates that the architecture and plasticity of an interphase chromosome is genetically specified by repetitive DNA-based structural codes that are “printed” in the linear genome. These structural codes would ensure a topological structure of interphase chromosomes can be created in a reproducible fashion.

## 3. Implications of the Model

The essential feature of the proposed structure that is of biological interest is that the spatial association of DNA repeats determines chromatin organization in interphase chromosomes. The RP-based repeat association is a driving force that causes site-specific chromatin folding and crosslinking. As a result, an internal order of chromatin organization is created within the seemingly amorphous interphase chromosomes. This internal order is a fundamental structural characteristic of interphase chromosomes. The suggestion of RP-based chromatin organization implies that the assembly and disassembly of RAs are a determining factor of structural changes of interphase chromosomes. It has been well established that interphase chromosomes are dynamic; their organization constantly changes in a non-random manner [21]. Based on this model, I suggest that the dynamic alteration of interphase chromosome structures is governed by the formation and disruption of specific RAs. The chromosomal dynamics are known to be regulated by chromatin remodeling complexes [22]. Interestingly, Alu repeats are in the genomic binding site for human ISWI-containing chromatin remodeling complexes [23]. It is tempting to imagine that the complex remodels chromosomes via Alu-containing RA nodes.

If the suggested model is correct, it predicts that a given set of RPs leads to a specific structure of an interphase chromosome and that the change of RPs leads to correspondent alterations of chromosomal structures. Under this conceptual framework, I suggest that, for a specific chromosome, its mitotic (e.g., metaphase) and interphase states are intrinsically related forms of chromatin organization. These organizational forms are specified by the repetitive DNA elements in each chromosome. The interphase chromosome is the relaxed form of its mitotic counterpart. The relaxation is due to the disassembling of specific RAs in interphase nuclei. Conversely, the mitotic chromosome is the condensed form of its interphase counterpart. The condensation is largely, if not totally, driven by the massive increase of DNA repeats assembling into RAs in the mitotic nuclei. In other words, the switching between these forms of chromosomal organization is governed by repeated DNA elements. This suggested structural correlation of interphase and mitotic chromosomes is consistent with the observation of the apparent organizational correspondence between them [1,24]. Similar to the mitotic chromosomal skeleton formed by the assemblies of tandem and dispersed DNA repeats [10], the tandem and dispersed repeat assemblies (TRAs and DRAs) in the interphase nuclei also can be conceived to constitute the skeleton of interphase chromosomes. This skeletal structure determines the morphology of the interphase chromosome and specifies the internal interaction of chromatins within the interphase chromosome.

Recent studies led to the insights that co-regulated transcription may occur among different genes that are spatially clustered in the giant loop field of the nuclear space, as a result of the long-range interactions of chromatins [25,26]. Although there is no direct evidence yet, I conceive that the model of RA-organized chromatin network may provide a structural platform for such co-regulation of gene transcription. In this structure, RPs are expected to generate chromatin loops and chromatin-chromatin interactions and thus lead to RA-based compartmentation of chromatins in the interphase chromosomes. Conceptually, RA-directed interphase chromosomal compartmentalization can create functional domains of the chromosomes. In these domains, genes that are separated in the linear genome but in the proximity of the participating DNA repeats are clustered around RA nodes, and are therefore co-regulated by the same pool of transcriptional factors. The details of this idea will be developed sufficiently in a separate manuscript.

As I have suggested [9], the physical distance between repeats is expected to constrain the RPs. On the same chromosome, the shorter the linear distance between two homologous repeats, the higher the probability they undergo RP. Such a mechanism of distance-regulated RPs generates a critical spatial constraint that governs the folding of linear chromatin. If this hypothetical mechanism indeed operates, it predicts that chromatins in interphase chromosomes are folded in a way that proximal segments of chromatin are preferably associated. This prediction is consistent with the model of fractal globule organization of interphase chromosomes, proposed recently based on chromatin interaction studies [7].

In addition to the suggested role of RPs and RA formation among *cis* repeats in specifying the internal organization of interphase chromosomes described above, repeat interactions may also participate in inter-chromosomal interactions. Limited intermingling of adjacent interphase chromosomes have been reported [12]. It is possible that RPs occur among *trans* repeats on different chromosomes. Such *trans* RP-mediated inter-chromosomal interactions may generate a critical molecular constraint that specifies the relative positions of the chromosomal territories (CT) of different chromosomes. Two chromosomes with many RPs are expected to be spatially close to one another in the nuclear space. Under this framework, the spatial position of a specific interphase chromosome is specified by its total RPs with other chromosomes in the same nucleus. It is intriguing to note that homologous CTs are often not spatially associated with one another in somatic cells. This indicates that RPs do not favorably occur between homologous chromosomes. One possibility is that RPs predominantly form within the same chromosome because of spatial and steric constraints. The intrachromosomal RPs may introduce structural barriers that limit the formation of interchromosomal RPs. Such a restriction is probably important for creating and maintaining the internal chromatin order of chromosomes.

In the organized chromatin mesh of interphase chromosomes, a large number of RA-formed nodes are formed to anchor and crosslink chromatins. Numerous DNA repeats are expected to be involved in this organization. This function of repetitive DNA, if validated, may provide a simple answer to the following question that has been puzzling us for last few decades: why are there so many repetitive DNA elements in the genomes?

## 4. Conclusions

Various models have been proposed to provide a structural and/or organizational description of interphase chromosomes in the nuclear space. However, the understanding of molecular mechanisms by which the interphase chromosomes are specified is still largely lacking. The repetitive DNA-mediated chromatin mesh proposed here emphasizes the role of repetitive DNA as a structural code for the interphase chromosome organization. The major structural features of the model, including the RA-formed nodes and chromatins around the nodes, are consistent with many of the published observations and agree with the key elements of previous lattice models [4]. The new contribution of the current model is that, in addition to a description of the internal organization, it suggests that this internal organization of chromatins in interphase chromosomes is determined by pre-specified chromatin folding that is mediated by RP and RA formation. This RP-based specific molecular mechanism suggests that interphase chromosomes are highly organized internally, despite their apparently amorphous appearance under microscopes. Although the same interphase chromosome may appear with different morphologies in different cells, a relatively stable topological structure is expected to be maintained by RPs and RAs. The mesh of the same chromosome is expected to be topologically similar in the same type of cells. It is important to conceive, however, that cells in interphase may have different intracellular states and thus different RP and RAs. Accordingly, it is reasonable to envision that there are different sub-types of interphase chromosome organization. Each of the sub-types would be specified by a defined group of RPs and RAs.

For now, the general scheme of the RP-based mesh organization of interphase chromosomes, as described above, must be regarded as speculative. Much remains to be learned before the RP-organized interphase chromosomal structure can be described in detail. What is the specific set of DNA repeats that determine the organization of a given interphase chromosome? How do specific repeats find their pairing partners among many candidates? How are pairing repeats assembled to RAs? Do other *trans* macro-molecules (e.g., proteins, RNAs) facilitate the assembly of RAs? If yes, how do they do so? Do assembling and disassembling of RPs drive the structural dynamics of the interphase chromosomal lattice?

In spite of these uncertainties, I suggest that the proposed mesh structure may shed light on the molecular basis of chromatin organization in interphase chromosomes. The hypothesis that I suggest here is that pairing (or association) of homologous DNA repeats creates a molecular order within the seemingly amorphous interphase chromosomes. The proposed chromatin mesh is formed because of this molecular order.
